# Unveiling the therapeutic promise: exploring Lysophosphatidic Acid (LPA) signaling in malignant bone tumors for novel cancer treatments

**DOI:** 10.1186/s12944-024-02196-9

**Published:** 2024-06-28

**Authors:** Yichen Qi, Yukai Wang, Jinping Yuan, Yufei Xu, Haili Pan

**Affiliations:** 1 Huankui Academy, Nanchang University, Nanchang, 330031 China; 2 School of Life Sciences, Nanchang University, Nanchang, 330031 China; 3 The First Clinical Medical College, Nanchang University, Nanchang, 330031 China; 4 Neurological Institute of Jiangxi Province, Department of Neurology, Jiangxi Provincial People’s Hospital, The First Affiliated Hospital of Nanchang Medical College, Nanchang, 330006 China; 5 Department of Neurology, Xiangya Hospital, Central South University, Jiangxi Hospital, National Regional Center for Neurological Diseases, No. 266 Fenghe North Avenue, Honggutan District, Nanchang, Jiangxi 330038 P. R. China

**Keywords:** Bone cancer, Bone metastasis, Lysophosphatidic acid, Cancer-induced bone pain

## Abstract

Malignant bone tumors, including primary bone cancer and metastatic bone tumors, are a significant clinical challenge due to their high frequency of presentation, poor prognosis and lack of effective treatments and therapies. Bone tumors are often accompanied by skeletal complications such as bone destruction and cancer-induced bone pain. However, the mechanisms involved in bone cancer progression, bone metastasis and skeletal complications remain unclear. Lysophosphatidic acid (LPA), an intercellular lipid signaling molecule that exerts a wide range of biological effects mainly through specifically binding to LPA receptors (LPARs), has been found to be present at high levels in the ascites of bone tumor patients. Numerous studies have suggested that LPA plays a role in primary malignant bone tumors, bone metastasis, and skeletal complications. In this review, we summarize the role of LPA signaling in primary bone cancer, bone metastasis and skeletal complications. Modulating LPA signaling may represent a novel avenue for future therapeutic treatments for bone cancer, potentially improving patient prognosis and quality of life.

## Introduction

Cancer is defined by the unbridled proliferation and dissemination of aberrant cells. These anomalous cells can coalesce to form tumors, which manifest as abnormal tissue masses. Tumors may exhibit either malignancy or benignity. Malignant tumors can infiltrate neighboring tissues and metastasize to distant sites via the circulatory and lymphatic systems [1]. Cancer poses a significant threat to global public health. In 2022, nearly 20 million new cases of cancer and close to 10 million cancer-related deaths occurred worldwide. Demographic projections suggest that by 2050, the annual incidence of new cancer cases will soar to 35 million, marking a 77% surge from 2022 [2]. One major contributing factor to the high mortality rate is late-stage diagnosis. Despite efforts, early detection of cancer remains challenging, as cancers are typically asymptomatic in their initial stages, complicating timely diagnosis. Additionally, accurate and precise diagnosis is imperative for mitigating biases and addressing challenges associated with rare and complex conditions [3]. Current cancer treatment faces several challenges, including drug resistance, side effects, and obstacles in immunotherapy. Improving patient survival rates and extending survival time necessitate concerted efforts and further research to validate interventions. Factors such as tumor heterogeneity, individual patient differences, treatment accessibility, and economic burden also influence treatment outcomes. In summary, personalized and precise treatments tailored to individual patients are imperative [4]. Bone tumors specifically originate from abnormal cell growth within bone tissue. Malignant bone tumors, including primary bone cancer and bone metastasis, are prevalent and malignant tumors with a poor prognosis [5]. Primary bone cancers, such as osteosarcoma, Ewing sarcoma, and chondrosarcoma, are relatively rare. The skeleton is a common site of metastasis for most human cancers, including lung cancer, breast cancer and prostate cancer [5]. Bone cancer is often accompanied by skeletal complications, such as pathological pain and other skeletal-related events [6], which can significantly impact patients’ quality of life and survival rate. Therefore, effective treatments and therapies for bone cancer patients are needed.

Tumor progression is typically associated with the dysregulation of various biological pathways and mechanisms. Dysregulated activities can fuel malignant cell growth and differentiation, initiating tumor formation. For instance, autophagy, a process vital for intracellular homeostasis, has a dual role in cancer. In early tumorigenesis, autophagy serves as a tumor suppressor. However, in established tumors, its dysregulation can promote cancer cell survival amid metabolic stress and adverse microenvironments [7]. Furthermore, aberrant signaling pathways can lead to diverse physiological dysfunctions, with effects contingent on the specific pathway and context. Dysregulation of immunologic and oncogenic signaling pathways, for instance, can facilitate immune evasion, bolster tumor growth and hasten progression [8]. Clinical intervention often involves surgical resection, but when this is not feasible, pharmacological therapies are employed, albeit with the risk of developing drug resistance. The emergence of drug resistance is multifactorial. Apoptosis is a form of regulated cell death, which plays a vital role in the resistance of cancer cells to chemotherapy. Chemoresistance can stem from impaired cell cycle arrest, but certain drugs can synergize with chemotherapy by directly inducing apoptosis. Modulating these processes holds promise for reducing drug resistance and enhancing treatment efficacy. For instance, exosomes have been implicated in diminishing cancer cell susceptibility to chemotherapy-induced apoptosis. Additionally, increased cancer cell invasiveness likely contributes to drug resistance [9, 10]. Importantly, drug resistance is a primary driver of therapeutic failure in cancer treatment. Therefore, developing strategies to overcome this obstacle is paramount in oncology [11].

LPA is a bioactive glycerol phospholipid distributed in most tissues and body fluids. In recent decades, the significance of LPA in tumor progression has gradually been recognized since the first discovery of the upregulation of its metabolic precursor in the serum of patients with gynecological cancer [12]. LPA exerts its biological functions primarily by binding to LPARs, which are G protein-coupled receptors (GPCRs) that activate downstream signaling pathways by coupling to four Gα proteins (Gs, Gi/o, Gq/11 and G12/13). LPARs can be classified into two types: the endothelial cell differentiation gene (Edg) family of GPCRs and purinergic receptors. These genes were named LPAR1-6 according to the order in which they were first reported in the literature. The Edg family comprises LPAR1 (also known as Edg2), LPAR2 (also known as Edg4) and LPAR3 (also known as Edg7), while LPAR4 (also known as GPR23 or P2Y9), LPAR5 (also known as GPR9) and LPAR6 (also known as GPR87 or P2Y5) belong to the non-Edg family [13, 14]. Upon binding to appropriate receptors, LPA can activate mitogen-activated protein kinase (MAPK), phospholipase C (PLC), phosphoinositide 3-kinase (PI3K) and Ras homolog family member A (RhoA) pathways, which participate in various cellular processes [15], including the progression of bone cancer and related events.

Currently, a comprehensive review of the participation of LPA signaling in bone tumors is notably absent from the literature. In addition to primary bone tumors, the advent of bone metastasis represents a critical stage in which numerous malignant neoplasms infiltrate the body. As malignancies progress within osseous tissues, they precipitate diverse complications, such as bone cancer pain and fractures [5]. This review fills a critical gap by not only consolidating the multifaceted roles of LPA in the progression of primary bone cancer but also shedding light on its significance in bone metastasis and associated skeletal events in bone cancer. Furthermore, it meticulously explores the clinical implications of LPARs in these processes, encompassing the targeted modulation of LPARs and the exploitation of LPA signaling in combating cancer drug resistance and advancing disease treatment.

### The roles of LPA signaling in tumor progression

LPA has been identified in multiple cancer cell types, ascites fluid and tumor effusates cancer patients [16]. As a growth factor-like lipid mediator, LPA can stimulate the migration of cancer cells. Additionally, LPA production induced by autotaxin (ATX) transcription, LPA metabolic degradation and the expression of multiple LPARs can be changed in cancer cells, thereby affecting tumor growth [16]. LPA influences the development of tumor cells through specific signaling pathways, directly acting on cells and/or indirectly promoting the production of signaling molecules. Clarifying the role and mechanism of LPA in bone cancer may provide a proper therapeutic treatment for bone cancer.

#### Impact of LPARs expression on tumor development

LPARs are expressed in tumor cells as well as tumor-associated cells, serving as biomarkers for cancer, such as ovarian cancer [17], breast cancer [18], prostate cancer [19] and liver cancer [20]. Reinartz et al. isolated tumor cell spheroids, tumor-associated T cells and tumor-associated macrophages from the ascites of patients with high-grade serous ovarian carcinoma. Ascites is the form of late peritoneal fluid, contains a substantial number of tumor and immune cells that can interact with each other, generating and responding to mediators with properties that promote metastasis and suppress the immune system. LPAR1, LPAR2 and LPAR3 are expressed mainly in high-grade serous ovarian carcinoma cells, whereas LPAR5 and LPAR6 are predominantly expressed in tumor-associated tumor-associated T cells and macrophages [21]. These results suggested that LPA triggers different signaling pathways in ovarian carcinoma cells, tumor-associated T cells and tumor-associated macrophages.

The expression levels of LPARs in tumor cells can be upregulated or downregulated and may be involved in various cellular processes. Knockdown of LPAR1, LPAR3, LPAR4 or LPAR5 inhibited motility and invasion in human pancreatic carcinoma cells, whereas knockdown of LPAR6 had the opposite effect [22]. Okabe et al. detected increased expression of LPAR5 genes in hepatoma RH7777 cells derived from rat liver and adenocarcinoma RLCNR cells originating from the lung. Increased expression of the LPAR5 gene in hepatoma and adenocarcinoma cells correlates with its lack of methylation in the 5’ upstream region. Studies on the LPA’s effect on cell growth, LPA boosted the growth and movement of RLCNR and RH7777 cells. These findings indicate that heightened LPAR5 expression due to abnormal deoxyribonucleic acid (DNA) methylation could contribute to the growth advantage seen in tumor cells [23]. Tao et al. Noted significant lower expression of LPAR6 in clinical breast cancer tissues compared to paracancerous tissues. Knockdown of LPAR6 notably accelerated cell proliferation and migration in the ZR-75-1 cell line. Clinical parameter analysis revealed that patients with higher LPAR6 expression level had more favorable prognoses [24].

#### Interactions between LPA signaling and cancer-associated genes

Several genes play pivotal roles in cancer biology, orchestrating a myriad of cellular processes. Among these proteins, Ras, Myc, p53, Fas, and Fos are key players. Ras and Myc function as oncogenes, driving cell growth and survival, while p53 serves as a tumor suppressor. Additionally, Fas and Fos are involved in apoptosis and cellular signaling, respectively. Dysregulation of the expression and transcription of these genes is intricately linked to the occurrence and progression of tumors [25–28].

Despite their significance, there is limited research investigating the interactions between these genes and LPA signaling. Studies suggest that LPA signaling may counteract p53-dependent apoptosis by modulating p53 homeostasis via the activation of Akt, extracellular regulated protein kinase (ERK), or protein kinase A (PKA) in cancer cells or stromal cells within the tumor microenvironment [29]. For instance, in ovarian cancer cells, treatment with LPA led to a swift decrease in Fas expression on the cell surface, shielding these cells from apoptosis triggered by anti-Fas stimuli [30]. LPA also hindered the apoptosis enhanced by actin depolymerization, suggesting a protective role against immune cell attack and apoptosis induced by cytoskeleton-disrupting agents in epithelial ovarian cancer [31]. Moreover, in LPA-stimulated Rat-2 fibroblasts, cyclic adenosine monophosphate (cAMP) response element-binding protein (CREB) has been shown to upregulate Fos messenger ribonucleic acid (mRNA) levels [32]. However, experimental validations in cancer models are currently lacking.

#### LPA signaling pathways in tumor progression

The important role of LPA, a bioactive lipid mediator, in cell regulation has been recognized for years [33]. LPA binds to LPARs to activate downstream signaling pathways, playing functional roles in tumor progression. One such pathway is the PI3K/Akt pathway, known for enhancing cancer cell survival, motility, and proliferation [34]. For instance, LPA-LPAR1 signaling has been demonstrated to boost the proliferation and migration of esophageal squamous cell carcinoma (ESCC) cell lines through the PI3K/Akt pathway [35]. This finding suggested that targeting LPA-LPAR1 signaling could inhibit ESCC progression. In addition, LPA can influence the expression of tyrosine kinase receptors. By binding to the corresponding receptor, LPA can induce the upregulation of tyrosine kinase receptor expression through the MAPK, activator protein 1 (AP-1), early growth response protein 1 (Egr-1), as well as nuclear factor kappa-B (NF-κB) signaling axes. This, in turn, promotes the invasion of bladder cancer cells [36]. In addition, LPA serves as a critical mediator of cancer cell migration. Hao et al. have shown that LPA facilitates the migration of prostate cancer PC3 cells through LPAR1 activation and the subsequent engagement of downstream signaling pathways, including ERK and p38α MAPKs [37]. Bian et al. found that inhibiting mitogen-activated protein kinase kinase 1 (MEKK1) downstream pathways does not significantly affect LPA-induced ovarian cancer cell migration. LPA also triggers the translocation of focal adhesion kinase (FAK) to focal contact sites on the plasma membrane, a process that can be disrupted by pertussis toxin, dominant-negative H-Ras, or dominant-negative MEKK1. These findings suggest that the G(i)-Ras-MEKK1 signaling pathway plays a crucial role in LPA-induced migration of ovarian cancer cells by facilitating FAK redistribution [38]. Furthermore, LPA modulates intracellular signaling to promote ovarian cancer cell migration. By activating Rho-GTPases and their downstream effectors, such as Rho kinase and Rho-associated protein kinase (ROCK), LPA induces cytoskeletal rearrangement and cell morphology changes that promote cell migration. Additionally, LPA influences cell migration by regulating intracellular calcium ion levels and the expression of tight junction proteins [39]. These results highlight the complex network of signaling pathways activated by LPA and their collective role in promoting tumor progression.

Furthermore, LPA can affect cancer progression by promoting the production of signaling molecules. For example, LPA enhances the expression of vascular endothelial growth factor (VEGF)-C, supporting lymph angiogenesis in prostate cancer cells. They further demonstrated that calreticulin, a multifunctional chaperone protein, is critical for prostate cancer progression through mediating LPA-induced VEGF-C expression [40]. This finding suggests that LPA signaling is potentially involved in prostate cancer metastasis. Interestingly, LPAR5 has been shown to increase the level of intracellular cAMP, resulting in cancer suppression [41, 42]. These findings indicate that different LPARs can play distinct and sometimes opposing roles in cancer progression, further underscoring the complexity of LPA signaling in cancer. Moreover, evidence suggests that LPA may contribute to in breast cancer initiation, progression, and invasion by triggering the release of interleukin 6 (IL-6) and tumor necrosis factor (TNF)-α cytokines in MDA-MB-468 cells [43]. In summary, LPA enhances the invasive and migratory capabilities of cancer cells through various signaling pathways and molecular factors, endowing them with an elevated potential for metastasis. Metastasis represents the advanced stage of cancer progression, posing a significant challenge in developing strategies to impede cancer advancement. Further study is essential to fully grasp these mechanisms and their potential as therapeutic targets in cancer treatment.

### LPA signaling in primary malignant bone tumors

Bone tumors refer to the development of tumors within bone tissue, characterized by abnormal cell growth. In the context of cancer biology, somatic mutations in DNA result in altered cellular instructions, prompting cancer cells to undergo rapid proliferation, overriding normal regulatory mechanisms that would typically induce cell death. Consequently, an excessive accumulation of cancer cells occurs, potentially culminating in the formation of a tumor mass [44]. The occurrence of tumors involves a complex interplay between the tumor cells, the microenvironment, and immune cells. The genesis of bone tumors involves aberrant cell growth within bone tissue, originating from bone-forming cells (osteoblasts), cartilage-forming cells (chondrocytes), or other cell types [45]. The latest World Health Organization (WHO) summarized the classification of bone tumors [46]. The primary malignant bone tumors often include bone sarcomas like Ewing’s sarcoma, osteosarcoma, and chondrosarcoma [47]. Despite their rarity and high mortality rates, these diseases receive limited attention. Current studies predominantly explore the correlation between LPARs and cancer progression. However, the specific mechanism underlying the pathogenesis of primary bone cancer remains unclear. Current treatments for cancer include multiagent induction chemotherapy, surgical resection, and radiotherapy [48]. Despite advancements in surgical techniques and chemotherapy in recent decades, the prognosis of patients is still unsatisfactory. The recurrence and prognosis of this disease still depend on the occurrence of metastases. Therefore, targeting the factors involved in tumor progression, invasion and migration may be essential for curing this disease. Identifying new therapeutic targets and developing more effective treatment strategies could enhance the prognosis and life quality of patients with primary bone tumors. Here, we reviewed the impact of LPA signaling on the two most prevalent primary bone cancers: osteosarcoma and Ewing’s sarcoma.

#### Osteosarcoma

Osteosarcoma, a primary malignant tumor which originates from mesenchymal tissue, commonly affects children and adolescents. The function of LPARs in tumor cells has been studied in different cell lines in recent years. Okabe et al. noted mutations in both LPAR1 and LPAR3 in human osteosarcoma cells (MG-63 cells), suggesting their involvement in the pathogenesis of human osteosarcoma cells [49]. Their team further investigated the motility and invasion of LPAR3-knockdown HOS osteosarcoma cells. They observed a significant inhibition of cell motility compared to control groups, suggesting that LPAR3 positively regulates the cell motility in osteosarcoma cells [50]. LPAR1, LPAR2 and LPAR3 signaling were demonstrated to promote motility and invasion in osteosarcoma cells. Takagi et al. confirmed the high level of LPAR1 expression in 6 osteosarcoma cell lines and determined that LPA-induced invasion was impeded in LPAR1 knockout cell lines, emphasizing the essential role of the LPA-LPAR1 axis in osteosarcoma cell invasion [51]. In addition, LPAR2 has been implicated in the acquisition of malignant characteristics during MG-63 osteosarcoma cell progression [52]. Endothelial cells, crucial elements of the tumor microenvironment, modulate MG-63 cell motility through the activation of LPAR2 and LPAR3 signaling [53]. Further investigation is warranted to fully comprehend these mechanisms.

**Fig. 1:**
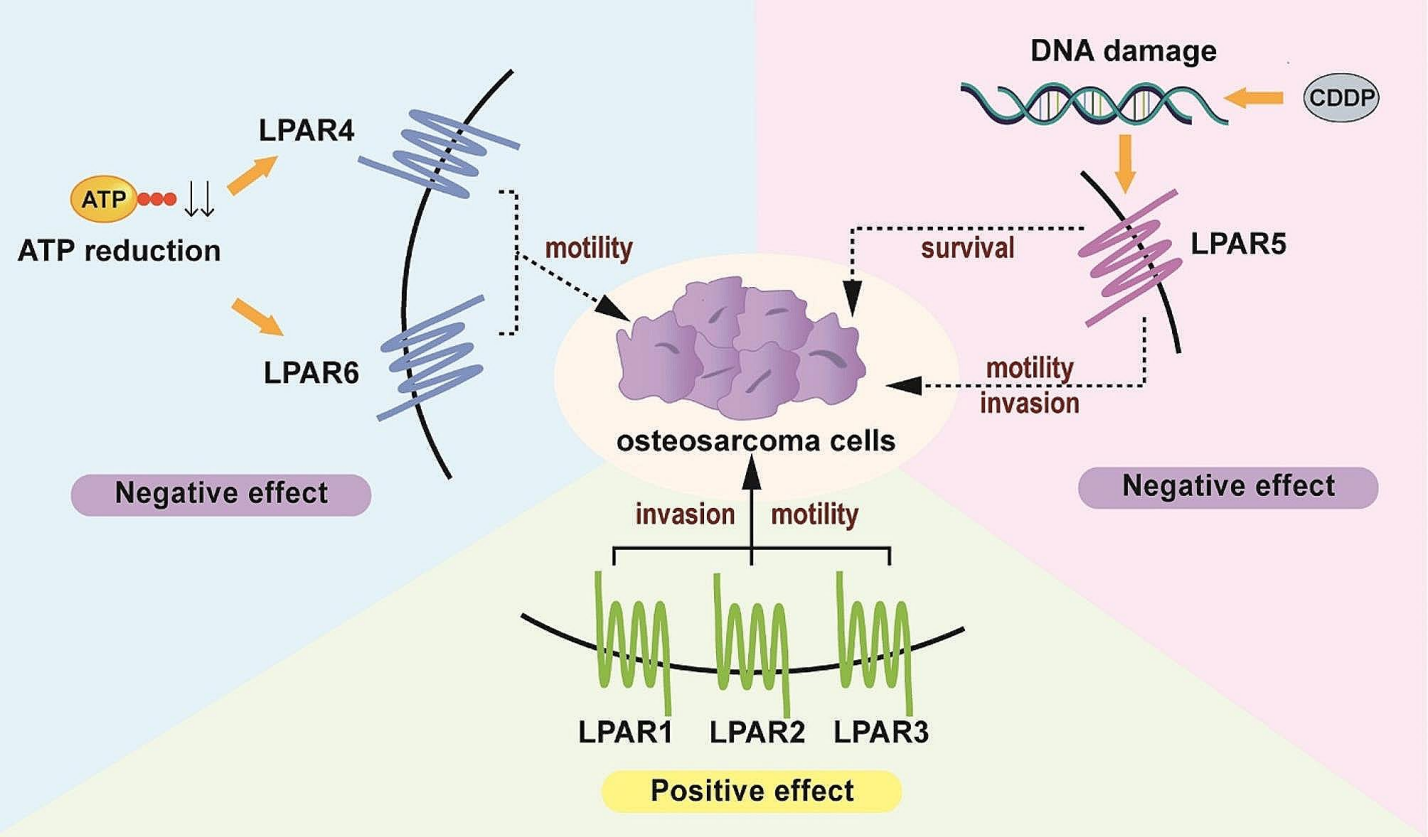
Positive and negative effects of LPARs in osteosarcoma. The specific mechanisms of LPARs in the pathogenesis of osteosarcoma are not yet fully understood. Current research on the relationship between LPARs and osteosarcoma progression primarily focuses on the regulation of cell functions, such as invasion and motility. LPAR1, LPAR2, and LPAR3 promote the invasion and migration of osteosarcoma cells. Under specific conditions, other LPARs have also shown some effects. For example, when CDDP, an anticancer drug known to cause DNA damage, was administered to osteosarcoma cells, it activated LPAR5 signaling, which led to a decrease in cell survival. Moreover, the knockdown of LPAR5 in osteosarcoma cells resulted in an increase in both motility and invasion, suggesting a negative correlation between these activities and LPAR5. When the intracellular ATP concentration was down-regulated, the survival of osteosarcoma cells treated with CDDP was improved by LPAR4 and LPAR6 knockdown. This result suggested that LPAR4 and LPAR6 may act as negative regulators of osteosarcoma cell motility

However, LPAR5 may have a negative effect on osteosarcoma [53, 54]. Cisplatin (CDDP), which is used for anticancer treatment, can cause damage to the DNA of cancer cells. Minami et al. reported that endothelial cells activating LPAR5 signaling in osteosarcoma cells led to reduced cell survival in response to CDDP [53]. The activation of matrix metalloproteinases (MMPs) can promote the invasion and migration of tumor cells [55]. Studies have shown that the expression of MMP-2 is increased in LPAR5-knockdown osteosarcoma cells and that both motility and invasion are promoted, suggesting that the motility and invasion of osteosarcoma cells are related to LPAR5 [53, 54]. Kurisu et al. conducted a study evaluating the effect of LPA signaling on osteosarcoma cells with reduced adenosine triphosphate (ATP) levels, which can result in necrosis and apoptosis. They used ethidium bromide to reduce the intracellular ATP level and found that LPA increased invasive activities in the treated cells, and LPAR4 and LPAR6 knockdown enhanced the survival of MG-63 cells treated with CDDP. These results suggest that LPAR4 and LPAR6 negatively regulate the osteosarcoma cell motility [56]. Therefore, the dysregulation of LPARs could significantly impact the advancement of osteosarcoma by regulating cell migration, invasion and metastasis (Fig. 1).

#### Ewing’s sarcoma

Ewing’s sarcoma, an aggressive bone and soft tissue cancer affecting children and young adults, constitutes approximately 2% of childhood cancers and ranks as the second most frequent bone cancer in children [57]. Although it was initially diagnosed as osteosarcoma, its unique properties make it generally considered a separate bone tumor [57]. There are few studies concerning Ewing’s sarcoma. Early studies have shown that Ewing family tumor cell lines express high levels of LPARs [58]. Nevertheless, Willier et al. reported that the LPA-generating enzyme lipase member 1 is almost exclusively overexpressed in Ewing’s sarcoma, which is a malignant neoplasm with high invasiveness, suggesting that LPA might be involved in the induction of invasive activities in primary bone tumors [59].

#### Other primary bone cancers

Limited research currently exists on the role of LPA in other primary bone cancers. For instance, studies on chondrosarcoma are scarce. Research has focused on elucidating the impact of 2-carba-cyclic phosphatidic acid on inflammatory and catabolic responses in human osteoarthritis synoviocytes and chondrosarcoma SW1353 cells in vitro [60]. Notably, the inhibitory effects of 2-carba-cyclic phosphatidic acid on MMP-1 and MMP-3 production in synoviocytes and MMP-13 production in chondrosarcoma SW1353 cells were not mediated by LPAR1 [60]. In the case of chordoma, a rare bone tumor with malignant potential, no studies have yet established a connection between LPA and its pathophysiology. Further research is required to investigate this potential association.

### LPA signaling in metastatic bone tumors

Metastasis is a complex process involving multiple steps and begins with increased invasiveness of tumor cells and detachment from the primary site. Subsequently, tumor cells can invade surrounding tissues and blood vessels, leading to distant organ dissemination and adaptation to foreign microenvironments [61]. Cancer cells migrate to the osseous tissue via the bloodstream or the lymphatic system, where they interact with bone cells and the bone microenvironment, resulting in the development of new tumor sites within the bone marrow cavity. The process of bone metastasis is characterized by several stages: tumor cell intravasation, circulation survival, extravasation at secondary sites, and subsequent tumorigenesis coupled with angiogenesis. This progression entails complex interactions with osteoclasts, promotion of angiogenesis, and modulation by a spectrum of cytokines and growth factors [62, 63]. Bone metastasis commonly occurs in late-stage cancer [64], and once it occurs, it becomes difficult to cure. However, the underlying mechanisms of bone metastasis remain unclear [65]. The “vicious cycle” theory is a widely accepted mechanism for bone metastasis that involves the interaction between tumor cells and bone cells, leading to the disruption of normal bone homeostasis and the promotion of tumor growth. LPA can influence this cycle, thereby affecting bone metastasis.

#### Communication between the bone microenvironment and tumor cells in bone metastasis

The microenvironment plays a crucial role in the process of tumor metastasis. At the onset of metastasis, the microenvironment of the target organ undergoes adaptive changes to create a favorable environment for the colonization and growth of metastatic tumor cells [66, 67]. Tumor-derived factors within the microenvironment have been shown to contribute to skeletal metastasis. Prior to reaching the target organ, tumor cells release various factors that directly act on the target organ, altering the microenvironment to facilitate the survival and growth of metastatic tumor cells. These factors induce the stromal cells of the target organ to release a spectrum of molecules, including cytokines, metabolites, growth factors, glycoproteins and glycan-binding proteins that guide tumor cell colonization. Notably, cytokines derived from tumor cells and microenvironmental cells, such as C-X-C motif chemokine (CXCL-1), CXCL-2, IL-6, IL-1b and TNF-α, have been identified as stimulators of bone absorption and metastasis [68–71].

**Fig. 2:**
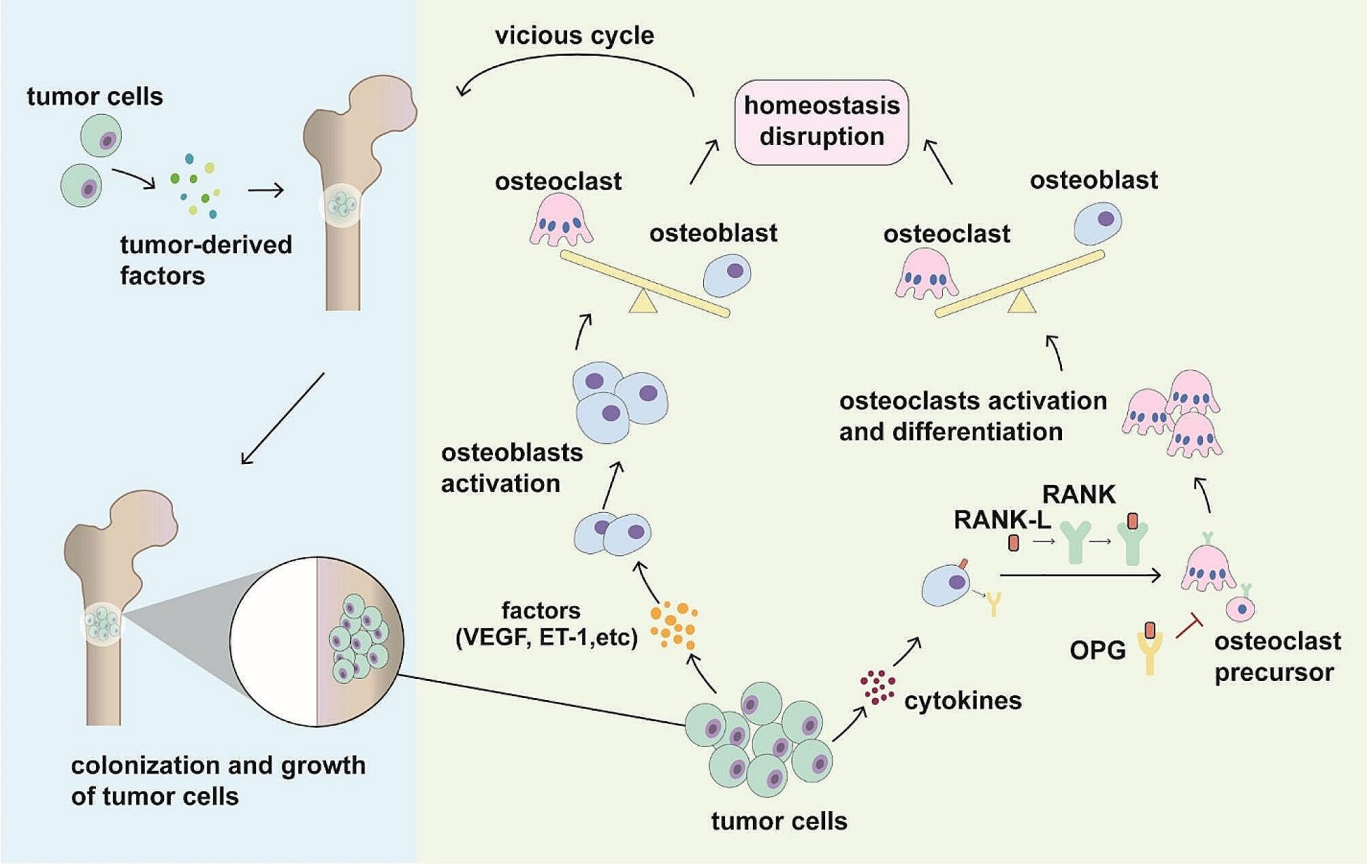
Communication between tumor cells and the bone microenvironment in bone metastasis. Tumor cells release diverse factors that directly influence the bone microenvironment, thereby altering the microenvironment to facilitate the survival and growth of metastatic tumor cells. This alteration enhances the colonization and proliferation of metastatic tumor cells. Once tumor cells colonize the bone matrix, they influence bone homeostasis through several processes. In the early stage of bone metastasis, tumor cells secrete various factors, such as VEGF and ET-1, stimulating the activity of osteoblasts. In addition, tumor-derived cytokines promote the RANKL/RANK/OPG axis, which enhances the differentiation and activation of osteoclasts. These processes disrupt bone homeostasis by affecting bone remodeling, which in turn promotes tumor growth and colonization in the bone microenvironment. Consequently, a vicious cycle is created

The bone microenvironment, which includes the extracellular matrix, blood vessels, and other bone cells, plays a crucial role in regulating bone growth and repair. Osteoblasts and osteoclasts are essential components of the bone microenvironment and work together to regulate bone remodeling and maintain bone homeostasis [72]. In the context of bone metastasis, however, this balance is disrupted, resulting in various phenotypes, including osteolytic, osteogenic and mixed patterns. Osteolytic bone metastasis is characterized by increased differentiation and function of osteoclasts and decreased osteoblast function. Conversely, osteoblastic metastasis involves increased osteoblast function and differentiation but decreased osteoclast activity [73, 74]. Therefore, excessively active osteoblasts or osteoclasts may be a characteristic of bone metastasis. In the early phases of bone metastasis, tumor cells secrete various factors like fibroblast growth factors, platelet-derived growth factor, VEGF and endothelin-1 (ET-1), stimulating the activity of osteoblasts [75]. In addition, osteoblasts can regulate osteoclast activity through direct cell-to-cell contact or through secretory proteins [72]. Receptor activator nuclear factor-κB (RANK) belongs to the TNF family and has been demonstrated to be a vital factor that drives cancer cell migration to bone. The differentiation and maturation of osteoclasts are induced by RANK and RANK ligand (RANK-L), thus promoting bone resorption [76, 77]. Osteoprotegerin (OPG), a decoy receptor of RANK-L, can prevent this process and inhibit the activation of osteoclasts [77–79]. OPG and RANKL bidirectionally regulate the activity of osteoclasts. The relative content of the two determines the activity of osteoclasts. For instance, in the case of osteolytic metastasis, following bone metastasis, cancer cells can secrete cytokines to increase the expression of RANKL while decreasing the expression of OPG in osteoblasts and other tumor-related cells, such as fibroblasts and immune cells. The balance between RANK-L and OPG is crucial for maintaining bone homeostasis. Disruptions to this balance can lead to increased osteoclast activity and bone resorption, contributing to conditions such as osteoporosis and bone metastasis. Increased RANK-L and decreased OPG enhance the effect of osteoclasts, which can increase bone destruction and cytokine release. Hyperactivation of osteoclasts exacerbates this effect and ultimately promotes bone metastasis [80, 81] (Fig. 2).

#### Involvement of LPA signaling in bone metastasis

LPA has been identified as a significant contributor to bone metastasis [82]. Several decades ago, researchers recognized a potential relationship between LPA and tumor metastasis. They discovered that metastatic tumor cells can activate platelets to release LPA and subsequently enhance tumor growth and bone destruction [83, 84]. Platelet-derived LPA acts on LPAR1-expressing tumor cells. LPAR1 can promote the release of cytokines such as granulocyte-macrophage colony-stimulating factor (GM-CSF), IL-6, IL-8, growth-regulated oncogene (Gro), and monocyte chemoattractant protein-1 (MCP-1) from MDA-BO2 breast cancer cells, which mediate osteoclast activation [85]. Nam et al. demonstrated that the enhanced expression of IL-8 by LPA may occur via the ROCK, protein kinase Cµ (PKCµ), PI3K, and NF-κB signaling pathways, while the enhanced expression of IL-11 might involve the protein kinase Cδ (PKCδ) signaling pathway in breast cancer [86]. In addition to similar findings in prostate cells, LPA was also reported to enhance osteoclastogenic cytokines, thereby increasing tumor growth and bone destruction. Conditioned medium from LPA-stimulated PC-3 prostate cells, enriched with osteoclastogenic cytokines, facilitated the formation of osteoclast. In particular, the levels of IL-6, GM-CSF, MCP-1, macrophage colony-stimulating factor **(**M-CSF), MCP-2, and insulin-like growth factor-binding protein (IGFBP) were greatly enhanced upon LPA stimulation [87].

ATX, also known as lysophosphatidylase D, can catalyze the conversion of lysophosphatidylcholine to LPA. Stromal ATX, which is also secreted by platelets, is highly expressed in bone metastases. These nontumor-derived ATXs are released due to tumor cell-induced platelet aggregation, leading to LPA production. Both in vitro and in vivo studies showed that ATX promoted the colonization of human breast cancer cells in the skeleton during bone metastasis [88]. In addition, in a clinical study, Shim et al. conducted immunohistochemical staining for ATX-LPA signaling-related proteins. They discovered that stromal ATX was highly expressed in bone metastases [89]. On the other hand, tumor-derived ATX controls bone metastasis through LPA-mediated osteoclasts activation. ATX expression enhanced human breast cancer MDA-B02 cell osteolytic bone metastasis in both in vivo and in vitro settings. The addition of purified LPA to lipid-depleted serum stimulated the complete differentiation of osteoclast precursors into osteoclasts with M-CSF/RANK-L in vitro [90]. Therefore, activation of the ATX/LPA axis in breast cancer cells plays a pivotal role in controlling the progression of bone metastasis by directly stimulating both cancer cells and osteoclasts.

**Fig. 3:**
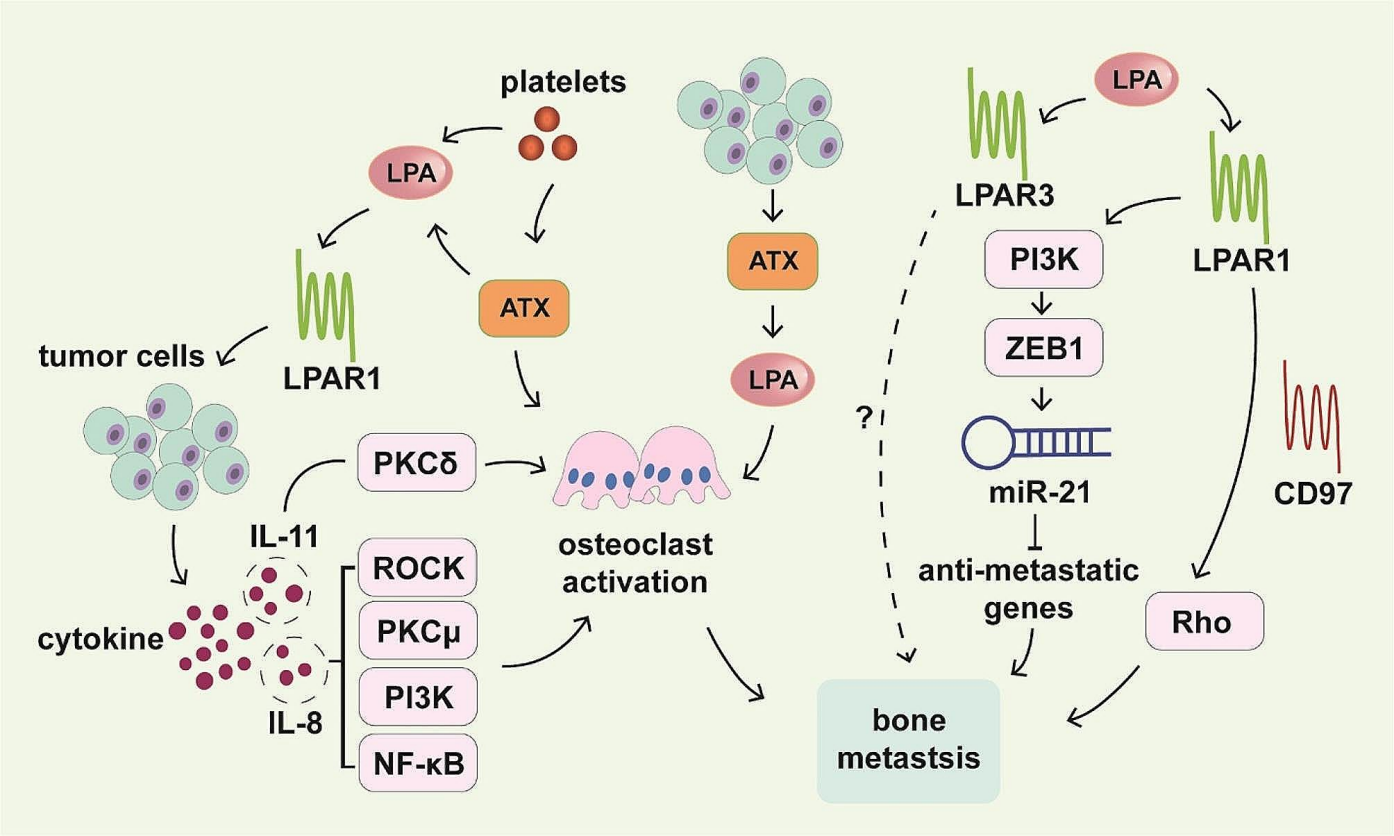
Involvement of LPA signaling in bone metastasis. LPA released by platelets acts on LPAR1, promoting cytokine production. These cytokines can stimulate osteoclast activation. Among the various cytokines secreted by breast cancer cells via LPAR1, the expression of IL-8 may be enhanced by LPA through the ROCK, PKCµ, PI3K, and NF-κB signaling pathways. Similarly, the increase in the expression of IL-11 might involve the PKCδ signaling pathway, which promotes both tumor growth and bone destruction. Furthermore, LPA could amplify osteoclastogenic cytokines. Stromal ATX is secreted by platelets. Nontumor-derived ATX promotes the early stage of bone metastasis and leads to the production of LPA. On the other hand, tumor-derived ATX regulates bone metastasis via LPA-dependent osteoclasts activation. In addition, LPAR1 and LPAR3 can facilitate skeletal metastasis. Upon acting on LPAR1, LPA can activate PI3K/AKT, leading to ZEB1 expression and downstream activation of miR-21 in breast cancer cells. Consequently, the expression of antimetastatic genes is inhibited, leading to metastasis. Interestingly, CD97 can form complexes with LPAR1 in cis, positively regulating LPAR1 and leading to increased LPA-dependent Rho signaling. Moreover, CD97 can heterodimerize with LPAR1, leading to LPA-initiated invasion and signaling. However, studies involving LPAR3 are relatively scarce, leaving the role of LPAR3 in bone metastasis unclear

In addition, David et al. demonstrated that competitive inhibitors of LPAR1 and LPAR3 can effectively suppress cell invasion and inhibit skeletal metastasis in mouse animal models. In particular, the increased antagonist activity of Debio 0719 at LPAR1 has been shown to greatly reduce the dissemination of tumor cells to bone both in vivo and in vitro. By blocking LPAR1 activity, the early stages of breast cancer cell bone metastasis to are impeded, not through angiogenesis and cell proliferation, but by inhibiting cell motility and invasion [91]. Their team further discovered that LPA can induce the LPAR signaling axis and activate downstream pathways. Through a combination of genetic manipulation and pharmacological interventions, they established that LPA’s proinvasive impact on triple-negative breast cancer cells relies on an LPAR1/PI3K/zinc finger E-box-binding homeobox 1 (ZEB1)/microRNA-21 (miR-21) activation cascade. LPA, via LPAR1, activates PI3K/AKT, initiating ZEB1 expression and subsequent miR-21 activation. This process suppresses the expression of antimetastatic genes (PTEN, PDCD4, and SPRY2), inducing cell migration, invasion and metastasis [92]. Interestingly, complement receptor 97 (CD97), an adhesion-linked GPCR, can interact in cis with and positively regulate LPAR1, enhancing LPA-dependent Rho signaling. The depletion of CD97 in PC3 prostate cancer cell lines reduced experimental bone metastasis without affecting subcutaneous growth in vitro. In addition, CD97 can heterodimerize with LPAR1, leading to LPA-initiated invasion and signaling [93] (Fig. 3).

### LPA signaling in cancer-related skeletal complications

#### Cancer-related bone pain

The complex interactions between bone cells and tumor cells not only drive tumor growth in bone but also cause pathological pain. Cancer-induced bone pain refers to the symptoms resulting from skeletal metastasis and pain caused by primary bone tumors, which may affect the physical function, quality of life and survival rate of patients [94, 95]. Schiwei et al. first reported a femoral cancer pain model in mice, in which pathological changes such as osteolysis and bone destruction, as well as behavioral changes such as spontaneous and touch-induced pain, were observed on the affected side of the model [96]. Bone cortex damage caused by bone tumors or invasion of the tumor itself can stimulate nerve endings in the periosteum and ultimately cause cancer-induced bone pain. Additionally, the secretion of inflammatory factors caused by an imbalance in the bone microenvironment sensitizes nerve fibers, leading to severe pain [95]. Although the mechanism underlying cancer-induced bone pain remains unclear, LPA signaling may affect cancer-induced bone pain by promoting tumor progression or disrupting bone homeostasis. Moreover, LPAR signaling can participate in the process of pain transmission.

Previous studies have indicated that LPARs could be potential targets for pain management [97–99]. Notably, the expression and activation of LPAR1 in dorsal root ganglion (DRG) neurons are associated with sensitizing C fibers, thereby inducing bone cancer pain [100]. LPAR1-mediated pathways have been shown to be crucial for the genesis of bone cancer pain. Inhibition of LPAR1 activity has been linked to mitigating bone cancer pain, possibly through the blockade of LPAR1/ERK signaling pathway or miR-329/LPAR1/ERK signaling. As a pleiotropic mediator, LPAR1 can modulate the ERK signaling cascade significantly, serving as a key regulator in bone cancer pain [101]. Other studies have shown that LPAR1 also participates in bone cancer pain regulation through Rho/ROCK signaling. Activation of the Rho/ROCK pathway can modulate bone cancer pain through P2 × 3 receptors [102].

**Fig. 4:**
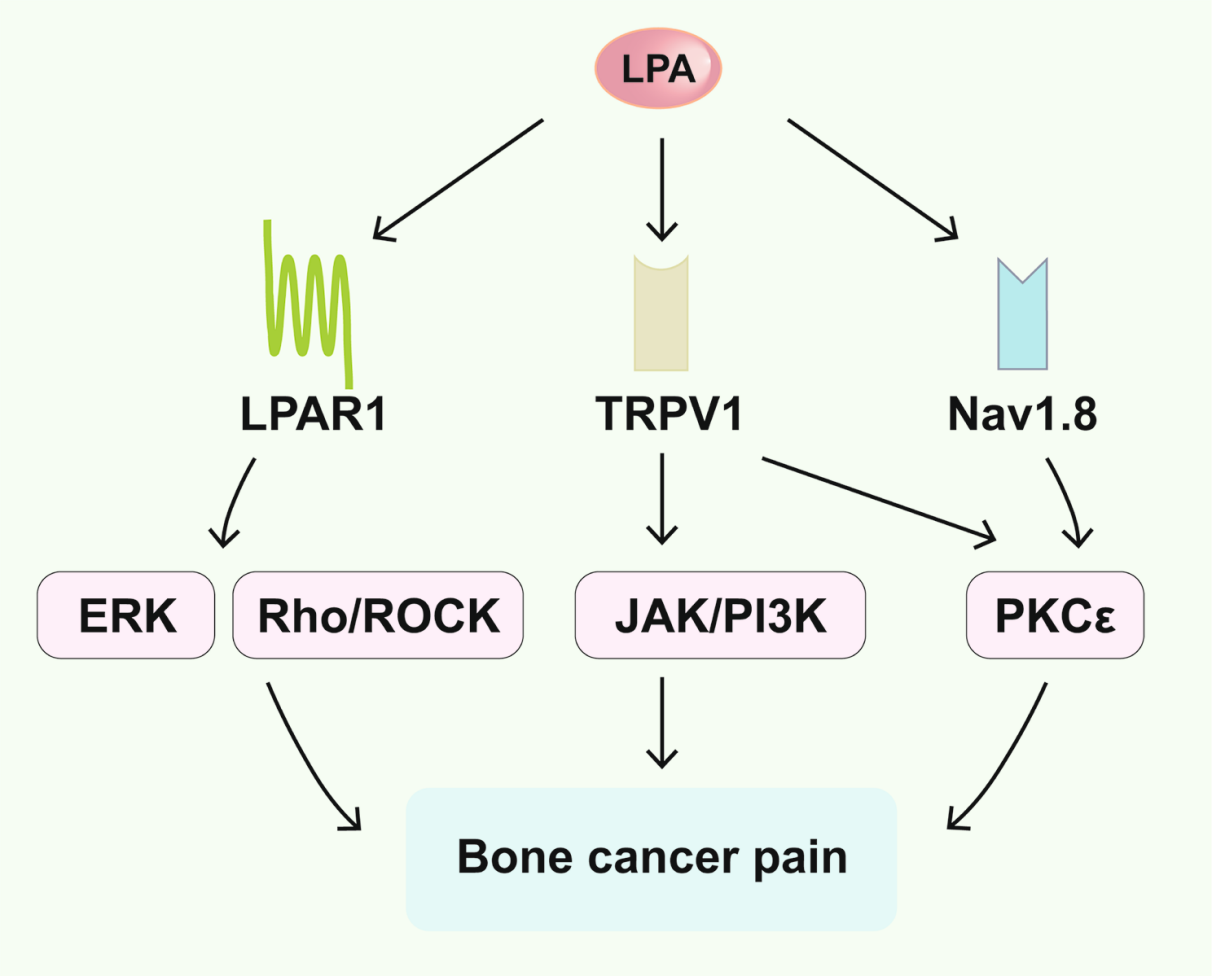
A brief overview of LPA signaling in bone cancer pain. LPA exerts its influence by directly activating LPAR1, which in turn triggers downstream cascades such as the ERK and Rho/ROCK signaling pathways, thus contributing to the onset of bone cancer pain. Furthermore, LPA sensitizes the TRPV1 receptor, subsequently activating either the JAK/PI3K signaling pathway or the PKCε signaling pathway, further fuelling bone cancer pain development. Finally, LPA enhances the Nav1.8 receptor, thereby activating the downstream PKCε signaling pathway and adding another layer to the pathogenesis of bone cancer pain

The interactions between LPA and ion channels are important for pain induction [103]. Transient receptor potential vanilloid 1 (TRPV1) is a non-selective cation receptor that is highly expressed in primary afferent neurons and senses peripheral nociceptive stimuli, leading to abnormal impulses in DRG neurons and resulting in hyperalgesia. LPA can enhance the activity of TRPV1 in nociceptors via a PKCε-dependent pathway [104]. Moreover, LPA signaling can generate IL-6, which contributes to the onset of bone cancer pain in rats through upregulating TRPV1 receptor function and activating the Janus kinase (JAK)/PI3K signaling pathway [104]. Pan et al. reported that intravenous injection of LPA can directly act on neurons and/or upregulate the expression of LPAR1 and Nav1.8 in the DRG and that the interaction between LPAR1 and Nav1.8 may contribute to the induction of bone cancer pain [105] (Fig. 4).

#### Skeletal-related events

Tumor cells exacerbate the imbalance between bone formation and bone resorption, promoting skeletal destruction and severe pathological manifestations [106]. Skeletal-related complications such as hypercalcemia, pathological fractures, spinal cord injury and uncontrolled pain requiring bone surgery and/or radiotherapy, are common complications of bone cancer. These events increase in frequency as bone cancer progresses and result in increased morbidity, mortality and healthcare costs [107].

LPAR1 has been shown to influence osteogenesis and bone development in vivo, but its absence may result in bone defects and osteoporosis [108]. LPA can partially rescue preosteoblast proliferation through the mitogen-activated protein kinase kinase 3 (MKK3)/MAPK/proliferating cell nuclear antigen (PCNA) pathway in ectonucleotide pyrophosphatase/phosphodiesterase 1 (Enpp1) deficiency-associated osteoporosis [109]. Moreover, LPA can stimulate osteoclast differentiation and induce the release of IL-6, which enhances bone destruction [110]. Some studies have reported that LPA can induce dendrite outgrowth in osteocytes and the secretion of cytokines that may promote fracture repair [111, 112]. However, the mechanisms underlying these pathological events remain unclear. Studies on the underlying mechanism by which LPA is involved in these events are relatively limited. Currently, palliative radiation therapy, chemotherapy, and surgery are common therapies for patients with skeletal-related events [113]. Skeletal-related events can be devastating complications for cancer patients. Once it occurs, it poses a severe threat to patients’ survival and quality of life. Further research could be performed to investigate the role of LPA in these complications, providing a novel strategy for their treatment.

### Clinical implications of LPA signaling as a therapeutic treatment

LPA not only contributes to tumor mechanisms but also has significant clinical value. LPA levels and LPAR expression may serve as potential diagnostic biomarkers for cancer patients [114]. Cao et al. conducted a case‒control study to investigate the plasma levels of LPA and found that LPA can serve as an biomarker for ovarian cancer progression and diagnosis [115]. Furthermore, Yu et al. identified metastatic tumors expressing LPAR1, LPAR2, and LPAR3 in the kidney, liver and pancreas; LPAR2 and LPAR3 in skeletal muscle; and LPAR2 in the cervical lymph node and heart [116]. In addition to diagnostic applications, therapy for malignant bone tumors has always been a focus of research. For primary bone tumors, comprehensive treatment with surgery as the primary approach has long been recommended in clinical practice. However, in recent years, limb-sparing therapy has gained popularity, and chemotherapy and radiotherapy have become important parts of treatment. For bone metastases, surgery is rarely an option. Most patients receive palliative care with chemotherapy and radiotherapy. Therefore, it is necessary to consider how to effectively implement chemotherapy and radiotherapy. Additionally, chemotherapy and radiotherapy indiscriminately attack host cells. The development of novel targeted drugs is also important.

#### Regulation of chemoresistance to anticancer drugs

In recent decades, researchers have been searching for effective and appropriate clinical therapies for cancer. Metastasis, a common outcome of malignant tumors, is closely related to high mortality rates [117]. To enhance patient survival rate and quality of life, chemotherapy combined with surgery has been applied to prevent complications and decrease patient mortality. CDDP is commonly used in chemotherapy for anticancer treatment because it can influence various cellular events and induce tumor cell death. However, when tumor cells are resistant to cisplatin, chemotherapy may fail. LPA has been found to modulate chemoresistance to anticancer drugs and plays a vital role in reversing cisplatin resistance. Ueda et al. reported that LPAR2 and LPAR3 agonists significantly increase the survival rate of lung cancer cells in response to CDDP [118]. Minami et al. reported that LPA treatment reduced osteosarcoma cell survival in combination with CDDP. Knockdown of LPAR5 in osteosarcoma cells resulted in a high cell survival rate in response to CDDP [53]. In addition, LPA exhibits promising potential for long-term CDDP treatment in cancer therapy. Ueda et al. also reported that the long-term survival rate of lung cancer cells treated with CDDP was elevated by an LPAR2 agonist [118]. Takahashi et al. reported that LPA treatment stimulated cell motility and invasive behaviors in fibrosarcoma cells exposed to long-term CDDP treatment. Knockdown of LPAR2 reduced these activities [119]. They also found that CDDP treatment increased LPAR2 and LPAR3 expression in osteosarcoma cells, where LPAR2 signaling played a role in enhancing cell motility, invasion, and colony formation under prolonged CDDP exposure [52].

#### Targeting LPA signaling for anticancer therapy

Targeting LPA signaling could serve as a treatment strategy for bone tumors. As early as 2006, it was reported that inhibiting the effect of LPA on LPAR1 could be a promising approach for treating bone metastasis [85]. In a separate study, BrP-LPA, acting as a pan-LPAR antagonist and ATX inhibitor, displayed the capability to impede the migration of human breast cancer cells (MDA-MB-231) in vitro, indicating potential to suppress cancer metastasis [120]. The potential value of LPAR antagonists for treating cancer has been recognized in recent decades. Debio-0719, a potent dual antagonist of LPAR1 and LPAR3, was identified for its ability to diminish pulmonary and bone metastases in murine 4T1 breast cancer cells while leaving the primary tumor size unaffected [91]. Additionally, Lin et al. reported that the use of an LPAR antagonist reduced lymphatic vessel density in prostate tumor cells, leading to decreased lymph node metastasis, underscoring the promise of targeting LPA signaling in prostate cancer therapy [40]. There have been ongoing clinical trials for drugs involving LPA signaling in the past decade [121–127] (Table 1). However, despite their clinical significance, clinical trials evaluating the efficacy LPAR antagonists in cancer treatment have not yet been performed. Corte et al. conducted a randomized, double-blind, phase 2 trial to test the impact of the LPAR1 antagonist BMS-986,278 on fibrotic disease [121]. Fibrosis and cancer share many common features and mechanisms, including the overproduction of growth factors and enhanced cellular senescence [128]. The results showed a promising safety profile and potential for treatment.

**Table 1:** Clinical trials and publications on LPAR and ATX targeted drugs in the past Decade

Compound	ClinicalTrials.gov ID	Disease	Phase	Publication
BMS-986,278 (LPAR1 antagonist)	NCT04308681	Idiopathic pulmonary fibrosis and progressive fibrotic interstitial lung diseases	Phase 2	[121]
BMS-986,020 (LPAR1 antagonist)	NCT01766817	Idiopathic pulmonary fibrosis	Phase 2	[122]
				[123]
				[124]
SAR100842 (LPAR1 antagonist)	NCT01651143	Diffuse cutaneous systemic sclerosis	Phase 2	[125]
GLPG1690 (selective ATX inhibitor)	NCT03798366	Diffuse cutaneous systemic sclerosis	Phase 2	[126]
GLPG1690 (selective ATX inhibitor)	NCT02738801	Idiopathic pulmonary fibrosis	Phase 2	[127]

#### The therapeutic potential of LPA in cancer immunotherapy

Tumor immunotherapy represents a form of cancer treatment designed to leverage the immune system’s ability to recognize and attack cancer cells. This therapeutic modality encompasses a spectrum of strategies, such as immune checkpoint inhibitors, adoptive cell transfer, and cancer vaccines. These approaches typically function by either activating the immune system or eliminating obstacles that impede its ability to target cancer cells [129]. LPA exerts diverse effects on immune cells and the tumor microenvironment, presenting a compelling avenue for investigation in immunotherapy. In metastatic ovarian cancer, tumor-derived LPA suppresses the production and signaling of type I interferons (IFNs), dampening the immune response against the tumor and facilitating tumor immune evasion and progression. The ATX-LPA axis serves as a crucial immunoregulatory pathway that diminishes protective type I IFN responses. Loss of ATX in ovarian cancer cells decreased LPA and prostaglandin E2 (PGE2) production at tumor sites, enhanced type-I IFN responses in tumor-associated dendritic cells, and augmented the efficacy of type I IFN inducers. Targeting LPA signaling pathways could potentially enhance type I interferon responses and boost antitumor immunity in ovarian cancer patients [130]. The LPA-LPA5 signaling axis is exploited by various cancers to inhibit T cell activation and function, highlighting the promise of targeting the LPA/LPAR pathway alongside anti- programmed death 1 (PD-1), anti-cytotoxic T-lymphocyte-associated antigen 4 (CTLA-4), or similar therapies to enhance immune function within tumors [131]. Melanoma-derived ATX repels tumor-infiltrating and circulating CD8 T cells via LPAR6, hindering their tumor infiltration and compromising anti-tumor immunity without affecting systemic T cell responses. Intratumoral ATX acts as a T cell repellent, unveiling a novel mechanism of the ATX-LPAR axis in promoting metastasis and suppressing CD8 T cell infiltration, offering potential therapeutic avenues [132].

LPA promotes the development of vascular networks within brain tumors, enhancing the efficacy of anti-PD-1 therapy. The RhoA/ROCK signaling pathway plays a crucial role in facilitating LPA-induced endothelial cell-cell adhesion, consequently regulating the expression of vascular cell adhesion molecule-1 (VCAM-1) and promoting increased lymphocyte infiltration into the tumor. Moreover, LPA aids in the delivery of exogenous IgG into brain tumors, further augmenting the anticancer effects of anti-PD-1 antibody therapy. These findings highlight the potential application of LPA-mediated modulation of vascular structure and function in the context of immunotherapy [133]. LPAR4 and LPAR6 are selectively expressed on high endothelial venule cells in lymph nodes. LPAR4 predominantly aids lymphocyte movement across high endothelial venules, with its absence leading to a significant lymphocyte accumulation in the endothelial cell layer. LPAR6 serves as a similar role, although its absence does not hinder lymphocyte movement to the same extent. While these findings may not directly relate to immunotherapy, they offer insights into the lymphocyte migration processes. Understanding the mechanisms governing the movement of these cells and their traversal of lymph node high endothelial venules holds significance for investigating the efficacy and mechanisms of immunotherapeutic interventions [134].

### Strengths and limitations

This review offers a comprehensive overview of the intricate role of LPA signaling in bone tumors. LPA influences tumor progression by activating downstream pathways and indirectly affecting tumor development through the modulation of factors such as cytokines and tumor-related genes. Notably, osteosarcoma, a prevalent primary bone tumor, is significantly impacted by the LPA signaling pathway, which affects the survival, invasion and motility of osteosarcoma cells. Moreover, the incidence of metastatic bone tumors surpasses that of primary bone tumors, prompting a focused exploration within this review on the influence of LPA on metastatic bone tumors, encompassing its effects on tumor cells, the bone microenvironment, and bone remodeling processes. In addition, as bone cancer pain and pathological fracture are common complications associated with bone tumors, this review also discusses the involvement of the LPA signaling pathway in these processes. From a clinical standpoint, LPA signaling has emerged as a potential inhibitor of tumor progression and a modulator of tumor drug resistance. Encouragingly, ongoing clinical trials investigating drugs targeting LPA signaling suggest their promise as safe and effective antitumor agents. Elucidating the correlation between LPA and bone cancer is highly promising for improving both the prognosis and the development of LPA-targeted drugs [135]. It is plausible that future therapeutic strategies harnessing LPA signaling could revolutionize cancer management [136].

However, it is imperative to acknowledge several limitations. The existing body of research predominantly relies on cell-based experiments, warranting the incorporation of a broader array of experimental methodologies to deepen our understanding of LPA signaling in bone tumors. Additionally, there is a pressing need for more comprehensive analyses elucidating the mechanisms by which LPA signaling contributes to the pathogenesis of malignant bone tumors, including a detailed exploration of its downstream effects. Despite promising results from preclinical models underscoring the therapeutic potential of LPA signaling, its application in clinical trials for tumors remains largely unexplored. Therefore, the initiation of further clinical trials to explore this potential is strongly advocated.

## Conclusion

In summary, this review illuminates the vital role of LPA signaling in primary bone cancer, bone metastasis and associated skeletal complications, with a specific emphasis on its implications in bone cancer. These findings not only enhance our understanding of prognosis but also drive the development of LPA-targeted drugs. Additionally, this review emphasizes the critical role of LPA signaling in mediating resistance to chemotherapy drugs, laying a robust foundation for innovative therapeutic approaches. These advancements offer hope for individuals with bone cancer, promising improved management and prognosis, ultimately leading to reduced mortality rates and a better quality of life.

## Data Availability

No datasets were generated or analysed during the current study.

## Abbreviations

LPA: Lysophosphatidic acid
LPAR: Lysophosphatidic acid receptor
GPCR: G protein-coupled receptors
Edg: Endothelial cell differentiation gene
PLC: Phospholipase C
MAPK: Mitogen-activated protein kinase
PI3K: Phosphoinositide 3-kinase
RhoA: Ras homolog family member A
ATX: Autotaxin
DNA: Deoxyribonucleic acid
ESCC: Esophageal squamous cell carcinoma
Egr-1: Early growth response protein 1
AP-1: Activator protein 1
NF-κB: Nuclear factor kappa-B
MEKK1: Mitogen-activated protein kinase kinase 1
FAK: Focal adhesion kinase
ROCK: Rho-associated protein kinase
ERK: Extracellularly regulated protein kinase
PKA: Protein kinase A
cAMP: Cyclic adenosine monophosphate
mRNA: Messenger ribonucleic acid
VEGF: Vascular endothelial growth factor
IL: Interleukin
TNF: Tumor necrosis factor
CDDP: Cisplatin
MMP: Matrix metalloproteinase
ATP: Adenosine triphosphate
CXCL-1: C-X-C motif chemokine
ET-1: Endothelin-1
RANK: Receptor activator nuclear factor-κB
RANK-L: Receptor activator nuclear factor-κB ligand
OPG: Osteoprotegerin
GM-CSF: Granulocyte-macrophage colony-stimulating factor
Gro: Growth-regulated oncogene
MCP-1: Monocyte chemoattractant protein-1
PKCµ: Protein kinase Cµ
PKCδ: Protein kinase Cδ
M-CSF: Macrophage colony-stimulating factor
IGFBP: Insulin-like growth factor-binding protein
ZEB1: Zinc finger E-box-binding homeobox 1
miR-21: microRNA-21
DRG: Dorsal root ganglion
TRPV1: Transient receptor potential vanilloid 1
JAK: Janus kinase
MKK3: Mitogen-activated protein kinase kinase 3
PCNA: Proliferating cell nuclear antigen
Enpp1: Ectonucleotide pyrophosphatase/phosphodiesterase 1
IFN: Interferons
PGE2: Prostaglandin E2
PD-1: Programmed death 1
CTLA-4: Cytotoxic T-lymphocyte-associated antigen 4
VCAM-1: Vascular cell adhesion molecule-1
